# Aging enhances serum cytokine response but not task-induced grip strength declines in a rat model of work-related musculoskeletal disorders

**DOI:** 10.1186/1471-2474-12-63

**Published:** 2011-03-29

**Authors:** Dong L Xin, Michelle Y Harris, Christine K Wade, Mamta Amin, Ann E Barr, Mary F Barbe

**Affiliations:** 1 Department of Physical Therapy, Temple University, 3307 North Broad St., Philadelphia, PA, 19140, USA; 2 Department of Anatomy and Cell Biology, Temple University School of Medicine, 3500 North Broad St., Philadelphia, PA, 19140, USA; 3 Department of Physical Therapy, Thomas Jefferson University, Philadelphia, PA, 19107, USA; 4 Office of the Provost, Pacific University, Hillsboro, OR, 97123, USA

## Abstract

**Background:**

We previously reported early tissue injury, increased serum and tissue inflammatory cytokines and decreased grip in young rats performing a moderate demand repetitive task. The tissue cytokine response was transient, the serum response and decreased grip were still evident by 8 weeks. Thus, here, we examined their levels at 12 weeks in young rats. Since aging is known to enhance serum cytokine levels, we also examined aged rats.

**Methods:**

Aged and young rats, 14 mo and 2.5 mo of age at onset, respectfully, were trained 15 min/day for 4 weeks, and then performed a high repetition, low force (HRLF) reaching and grasping task for 2 hours/day, for 12 weeks. Serum was assayed for 6 cytokines: IL-1alpha, IL-6, IFN-gamma, TNF-alpha, MIP2, IL-10. Grip strength was assayed, since we have previously shown an inverse correlation between grip strength and serum inflammatory cytokines. Results were compared to naïve (grip), and normal, food-restricted and trained-only controls.

**Results:**

Serum cytokines were higher overall in aged than young rats, with increases in IL-1alpha, IFN-gamma and IL-6 in aged Trained and 12-week HRLF rats, compared to young Trained and HRLF rats (p < 0.05 and p < 0.001, respectively, each). IL-6 was also increased in aged 12-week HRLF versus aged normal controls (p < 0.05). Serum IFN-gamma and MIP2 levels were also increased in young 6-week HRLF rats, but no cytokines were above baseline levels in young 12-week HRLF rats. Grip strength declined in both young and aged 12-week HRLF rats, compared to naïve and normal controls (p < 0.05 each), but these declines correlated only with IL-6 levels in aged rats (r = -0.39).

**Conclusion:**

Aging enhanced a serum cytokine response in general, a response that was even greater with repetitive task performance. Grip strength was adversely affected by task performance in both age groups, but was apparently influenced by factors other than serum cytokine levels in young rats.

## Background

Work-related repetitive strain injuries, also known as work-related musculoskeletal disorders, repetitive motion injuries and overuse injuries, are common injuries. They are injuries of the musculoskeletal and/or nervous systems caused by repetitive tasks, forceful exertions, vibrations, mechanical compression (pressing against hard surfaces), or sustained or awkward positions [1,2]. All of these disorders can be worsened by the repetitive actions of daily living [3]. Epidemiological studies have also demonstrated a relationship between advancing age and susceptibility to risk factors for musculoskeletal disorders [3-6].

Work-related repetitive strain injuries in the United States are estimated to cost over $61.2 billion annually. In 2006, the number of occupational injuries involving days away from work due to hand and wrist injuries were 47,020 and 56,250, respectively, while their incidence rates were 10.6% of 29.5% total in the upper extremity [1,2]. In the United Kingdom, 115,000 new cases of work-related repetitive strain injuries in the upper extremity were reported in 2006-2007 compared with 86,000 new cases in 2005-2006, and the number of people reporting such injuries rose from 374,000 to 426,000 [7,8]. Similar increases are reported by the French National Health Insurance Fund - Occupational Risks [9].

Our lab has reported that four circulating inflammatory mediators increased in patients with upper extremity repetitive strain injuries of no longer than 12 weeks and correlated with increasing symptom severity, including pain and decreased grip strength [10]. C-reactive protein was strongly correlated; interleukin 1 beta (IL-1β) and tumor necrosis factor alpha (TNFα) were moderately correlated, while IL-6 was fairly correlated with symptom severity. Identification of serum proteins that could be used as biomarkers for the presence of underlying inflammatory changes related to these disorders would be useful for both diagnostic purposes as well as for planning effective treatment of that inflammation.

We have developed a rat model of voluntary repetitive reaching and grasping with or without force, in which we found that the motor behavioral and physiological tissue changes that were similar to those seen in humans with repetitive strain injuries [11-24]. We have shown evidence of repetitive task-induced tissue injury, including myofiber fray, moth eaten muscle fibers, focal nerve compression and demyelination, and tendon collagen separation [12,15,16,21,24]. These injuries were associated with dose-dependent inflammatory responses, such as increased neutrophils, macrophages and inflammatory cytokines, in peripheral forelimb musculoskeletal and nerve tissues that were transient in rats performing low or moderate repetitive tasks, but higher and sustained with higher demand tasks [11-16,18,20,21,25]. Although only a transient inflammatory serum cytokine response was induced with our lowest demand task of low repetition and negligible force, serum cytokines were still elevated at 8 weeks at the termination of experiments examining the effects of performing a moderate demand task (high repetition, negligible force) [13]. However, we have yet to determine if this serum inflammatory response is sustained chronically with moderate demand tasks. Since serum cytokines are known to increase with many types of trauma and other conditions, including hypertension, cardiovascular disease, aging and obesity (reviewed in Carp et al, [26]), if such elevations of inflammatory mediators are additive, then repetitive strain injuries should be considered comorbities for these other conditions, and vice versa.

We have determined that several of these local tissue and serum inflammatory responses were associated with dose dependent declines in motor performance, such as grip strength, that were either transient or sustained (i.e. based on level of task and length of time performed) [12,13,15,16,18-22]. For example, increased circulating levels of tumor necrosis factor alpha (TNFα), macrophage inflammatory protein 2 (MIP2) and MIP3 correlated significantly with reduced grip strength in young rats that had performed a high repetition, negligible force food retrieval task for 8 weeks [13]. However, we have also reported that grip strength correlates strongly with nerve, muscle and tendon cytokine levels, decreased nerve conduction velocity indicative of nerve compression, and increased nociceptor-related neuropeptides in tendons and spinal cord dorsal horns [13,17,19,21], suggesting that many factors can affect grip strength. Therefore, we continue to investigate factors that contribute to declines in grip strength in our model.

While serum cytokine and chemokine response patterns to exercise of varying intensities and multiple organ trauma and fractures have been studied, exposure to more chronic, lower levels of injury are not yet well characterized. Also, the combined effects of both aging and performance of repetitive tasks need more evaluation since several epidemiological studies have shown a relationship between advancing age and susceptibility to musculoskeletal disorders [3,27]. Furthermore, chronic inflammation is a near-universal feature of aging mammals even in the absence of detectable disease or tissue injury, and can drive the pathogenesis of many age-related diseases. Several studies have reported age-related increases in several pro-inflammatory cytokines in serum, including IL-1α/β, IL-6 and TNFα [28-31]. These increases, in particular that of IL-6, correlate with and may even contribute to increased risk for incident disease, disability (including declines in hand grip strength) and mortality [32,33].

Using our rat model, we have recently shown that aged animals performing a repetitive upper extremity task show declining grip strength with continued task performance, increased forepaw mechanical hypersensitivity, decreased conduction velocity, increased TNFα in the median nerve at the level of the wrist and flexor digitorum muscles, and increased TNFα and IL-1β in the spinal cord [19]. However, we were unable to examine sera of that study due to the surgical procedure needed to examine median nerve conduction velocity in a rat, and thus were unable to determine if a systemic inflammatory response contributed to the declines in grip strength. While it appeared as if the aged rats had increased pathological responses to task performance than young rats in our prior studies, we examined only aged rats in that study. Nor has anyone else, to our knowledge, examined if aged animals performing repetitive tasks have similar or greater responses than young animals performing repetitive tasks with regards to serum cytokine levels or grip strength.

Therefore, we examined here for the first time if levels of 6 inflammatory-related cytokines in serum of aged and young rats performing the same voluntary high repetition, low force (HRLF) task for 12 weeks in which the upper limb shoulder and elbow joints were extended while the fingers and wrist are used to grasp a lever at 15% maximum pulling force and high repetition rate. Also, since this HRLF task differs somewhat from our previous high repetition and negligible force task, we also examined young rats at 6 weeks of HRLF task performance to ascertain if any serum changes observed in these and 12-week rats was a transient or sustained cytokine response. In serum, we examined 2 key pro-inflammatory cytokines (IL-1α and TNFα), 2 inflammatory chemokines (MIP2 and IFN-γ), and a potent anti-inflammatory cytokine (IL-10) that we have previously observed to increase in young rats performance repetitive tasks [13,18,22]. We also examined IL-6, a proteic cytokine with both pro- and anti-inflammatory properties (IL-6) [34], that did not increase in our prior studies examining young repetitive task rats but that is known to increase as a consequence of aging [32]. In addition, we compared a motor performance parameter, grip strength, with the systemic inflammatory response in order to evaluate the effects of the latter on motor function. Our hypotheses were that performance of repetitive upper extremity task would induce higher levels of circulating inflammatory cytokines as well as greater grip strength declines in aged rats than in young animals performing the same task, and that the levels of serum cytokines would correlate negatively with forearm grip strength.

## Methods

### Animals

All experiments were approved by the Institutional Animal Care and Use Committee in compliance with NIH guidelines for the humane care and use of laboratory animals. Studies were conducted on aged female and young adult female rats. A total of 55 rats were used: 23 aged, female Sprague-Dawley rats (14 mo of age at onset of task training; 18 mo of age at euthanasia) and 32 young female Sprague-Dawley rats (2.5 mo of age at onset of task training; 6.5 mo of age at euthanasia). Rats were housed individually in the central animal facility in transparent plastic cages in a 12-hour light: 12-hour dark cycle with free access to water.

Food restriction was necessary in this study to motivate rats to perform the repetitive task. Rats in the experimental group were food restricted to 5% of their normal weight during the training period and for the duration of the 12-week task. Although this was only a 5% restriction of food, since it has been reported that dietary restriction might retard age-related functional declines and pathogenesis in various organs [35-37], or might contribute to tissue catabolism, it was necessary to have food restriction only control groups in order to delineate the effects of food restriction on the variables (serum cytokines and grip strength). We also included trained only groups (food restricted and trained, but did not perform the task), since a pre-task training period was needed (described further below). Thus, in addition to the young and aged HRLF experimental task groups, 3 control groups were included in the design: normal controls (no food restriction, initial training or task performance), food restriction only controls (no initial training or task performance) and trained only controls (underwent food restriction and initial training, but no task performance) per age group.

**Figure 1:**
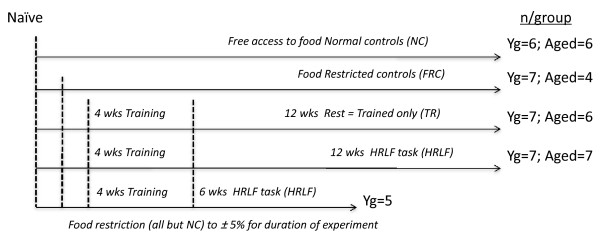
**Number of animals in each young or aged rat groups**. NC = normal controls with free access to food; FRC = Food restricted controls that were neither trained nor performed the task; TR = trained-only controls that were food restricted animals that were also trained for four weeks, and then rested for 12 weeks; HRLF = food restricted animals that were trained for four weeks, and then performed the high repetition low force task for 6 and 12 weeks.

Total of 43 rats (26 young rats and 17 aged rats) were food restricted to within 5% of their naïve weights or in comparison to the age-matched normal controls (Figure 1). Thirty-two of these rats (19 young and 13 aged) went through an initial training period of 4.0 ± 1.6 weeks (mean ± SEM), in which they were trained to perform the reaching and handle pulling task (described further below and in Figure 1). Nineteen (12 young and 7 aged) of these trained rats then went on to perform a high repetition, low force task for 6 week and 12 weeks (HRLF; described further below and in Figure 1). The remaining 13 trained rats (7 young and 6 aged), serving as trained-only rats (Trained), did not proceed past week 0 to the task regimen, but rested 12 weeks until euthanasia (Figure 1). The remaining 11 food restricted rats (7 young and 4 aged) were not trained, and served as food restricted controls (FRC; Figure 1). Twelve more rats (6 young and 6 aged) served as age-matched normal controls with free access to food (Figure 1). The normal controls rats did not undergo food restriction, training or task performance.

All rats were weighed weekly throughout the experiment and food adjusted accordingly. In addition to food pellet rewards (Bioserve, NJ), all rats received Purina rat chow daily. Trained and FRC rats received daily allotments of food pellets and rat chow matched to the HRLF rats. Normal control rats had free access to food. All rats were inspected weekly and again post-mortem for presence of illness or tumors in order to reduce confounders for serum cytokine increases. As a consequence, an additional 8 aged rats were eliminated from the study due to age-related health issues, such as renal failure, presence of tumors or mortality. To further reduce illness related confounders, additional sentinel rats were examined for presence of viral infections as part of the regular veterinary care (no viruses were detected).

### Behavioral Apparatus

Briefly, as described previously in detail [15,19] and as depicted previously [21], HRLF rats had to grasp the force handle at a target rate of 4 reaches/min and exert an isometric pull toward the chamber wall with a graded force effort of 15% ± 5% maximum voluntary pulling force. For this task, custom-designed force apparatuses were used (Custom Medical Research Equipment, Glendora, NJ). These apparatuses were integrated into an operant behavioral training system (Med Associates, Georgia, VT). A portal was located in the wall of the operant conditioning chamber at shoulder height (3.5 cm), so the shoulder had to be fully elevated and the elbow fully extended for the animal to reach through the portal to isometrically pull a custom-designed force handle attached to a force transducer (Futek Advanced Sensor Technology, Irvine, CA) connected to a 1.5 mm in diameter bar located 1.5 cm away from the portal entrance, outside the chamber wall. The bar was oriented at a 45-degree angle from vertical in the direction of pronation and was adjusted based on the preferred limb of the animal. The load cell was interfaced with a signal conditioner (Futek), which amplified and filtered the signal before it was sampled digitally at 100 Hz with Force-Lever software (version 1.03.02, Med Associates, St. Albans, VT). An auditory indicator cued the HRLF animals to reach every 15 seconds, i.e. a target rate of 4 reaches/min. A "reach" was defined as any time an experimental rat reached beyond and then withdrew the forepaw behind a line drawn 0.5 cm within the tube [20,25]. Some animals reached more frequently than the auditory prompts, suggesting that they did not rely on the auditory prompts to initiate a pull of the lever, and did not effectively learn that a food reward could only be obtained over a 5 second period 4 times per minute despite more frequent pulling of the lever. The rats also tended to have extra forearm movement reversals (extra fore-aft movements of the forelimb that occurred while reaching that were usually unsuccessful reaches, and thus an indicator of diminished fine motor coordination), as previously described and depicted [20,25]. However, since these extra reaches and extra forearm movement reversals contribute to total exposure, the software was calibrated to detect these extra movements and to include them in the mean reach rate. Thus "mean reach rate" was determined using the Med Associates and Force Lever software as the average number of all complete and incomplete reaches performed per minute; this was calculated on the last day of each week. The graded force effort of 15% ± 5% maximum voluntary pulling force fell between a minimum force criterion (12.5%, determined on the last day of training using Force Lever software, version 1.03.02, Med Associates) and a maximum force criterion (17.5%) for at least 50 ms. In grams, the average force was 28.05 g (range of 24.63 g to 34.48 g). If these force and time criteria were met within a 5 second cueing period, an indicator light turned on and a 45 mg purified formula food pellet (banana flavored; Bioserve, NJ) was dispensed into a trough located at floor height of the chamber in the wall panel adjacent to the aperture.

### Training Period

Prior to the initiation of the experiments, all rats were handled for 10 min/day for 2 weeks. Trained and task rats were food-restricted for a short period (no more than 7 days) to 5-15% of their naive weight (i.e., they lost no more than 5-15% of naïve body weight) to initiate interest in the food pellets, as described previously in detail [19]. After that first week, trained and task rats were given extra food chow and then maintained thereafter as closely as possible to within ± 5% of their naïve weight until euthanasia 15 weeks later. Thirty-two of the food-restricted control rats (19 young, 13 aged) went through an initial training period of 10-15 min/day, 5 days/week, for 4.0 ± 1.6 weeks (mean ± SEM), in which they were trained to perform the reaching and handle pulling task. As described above, 7 young and 6 aged rats were randomly selected to serve as Trained control rats, which did not proceed to HRLF task performance, but rested for 12 weeks while receiving a diet similar to the HRLF rats.

### HRLF task regimen

At the end of the training period, nineteen of the trained rats (12 young, 7 aged) were randomly selected to begin the HRLF task regimen at the target reach rate and force requirement (target of 4 reaches/minute; 15% maximum pulling force) for 2 hrs/day, 3 days/wk for 6 and 12 wks, thus serving as HRLF task rats. The task was divided into 4, 0.5-hr sessions separated by 1.5 hrs in order to avoid satiation. Rats were allowed to use their preferred limb to reach and their contralateral limb as a support limb, as needed, as depicted previously [21]. The side used to reach was recorded in each session. Thus, the animals were allowed to self-regulate their participation in task performance, making this a voluntary task.

### Grip Strength Analysis

Grip strength of the reach forelimb was tested as previously described using a grip strength meter for rodents (Stoelting, Wood Dale, IL) [15,19,21]. The test was repeated 5 times per forelimb, in a randomized fashion, and the maximum grip force (strength in grams) per trial was included in the statistical analysis. The person carrying out the testing was blind to treatment.

### Collection of Serum

Serum was collected from all rats. Following euthanasia (Nembutal, 120 mg/kg body weight), 18 hours after completion of the final task session, blood was collected by cardiac puncture using a 23-gauge needle put into 15 ml sterile tubes without any additives allowed to colt fro 30 min and centrifuged immediately at 1000 g for 20 min at 4°C. Serum was collected, flash-frozen, and stored at -80°C until analyzed.

### Measurement of serum cytokines and chemokines

The following cytokines and chemokines were analyzed in serum using a customized multiplexed sandwich ELISA system (Assay Gate, Ijamsville, MD): IL-1α, IL-6, IL-10, TNFα, MIP2 and IFN-γ. All samples were analyzed in duplicate in a blinded fashion, and as a batch in order to reduce potential inter-assay variability. Assay Gate protein assays have lower detection limits of 1.5 mg/ml for IL-1α, 6.3 mg/ml for IL-6, 5.4 mg/ml for IL-10, 3.1 pg/ml for TNFα, 1.1 pg/ml for MIP2, and 6.2 pg/ml for IFN-γ. IL-10 (lower detection limit of 0.8 mg/ml) was also tested using Aushon Searchlight Biosystem (Billerica, MA). Data are presented as pg cytokine/ml serum.

### Statistical Analyses

To determine the effect of age (young versus aged) and task performance (FRC and HRLF) on serum markers, two-way ANOVAs were used for the cytokine analyses. A Bonferroni post hoc analysis was then carried out comparing all groups (Normal controls, FRC, Trained, and HRLF) with each other so that any effects of food restriction or training could also be detected. In addition, for serum analyses, aged rats results were compared to the counterpart young rats (for example, 12-week HRLF aged versus 12-week HRLF young rats). Grip strength was compared similarly. A two-tailed Pearson's correlation test was used to correlate aged rat serum cytokine changes with their grip strength changes, and to correlate both young and aged rat serum cytokine changes, combined, with their grip strength changes. A *p *value ≤ 0.05 was considered significant.

## Results

### Mean reach exposure did not alter with age

The number of reaches per minute (including extra reaches and extra movement reversals as described and depicted previously [20,25]) did not significantly differ across weeks between young or aged HRLF rats (Young: 21.49 ± 2.63 mean ± SEM; Aged: 19.57 ± 1.98 mean ± SEM; p = 0.58), nor did total exposure (data not shown).

### Serum cytokines increase with training and HRLF task performance in aged rats only

**Figure 2:**
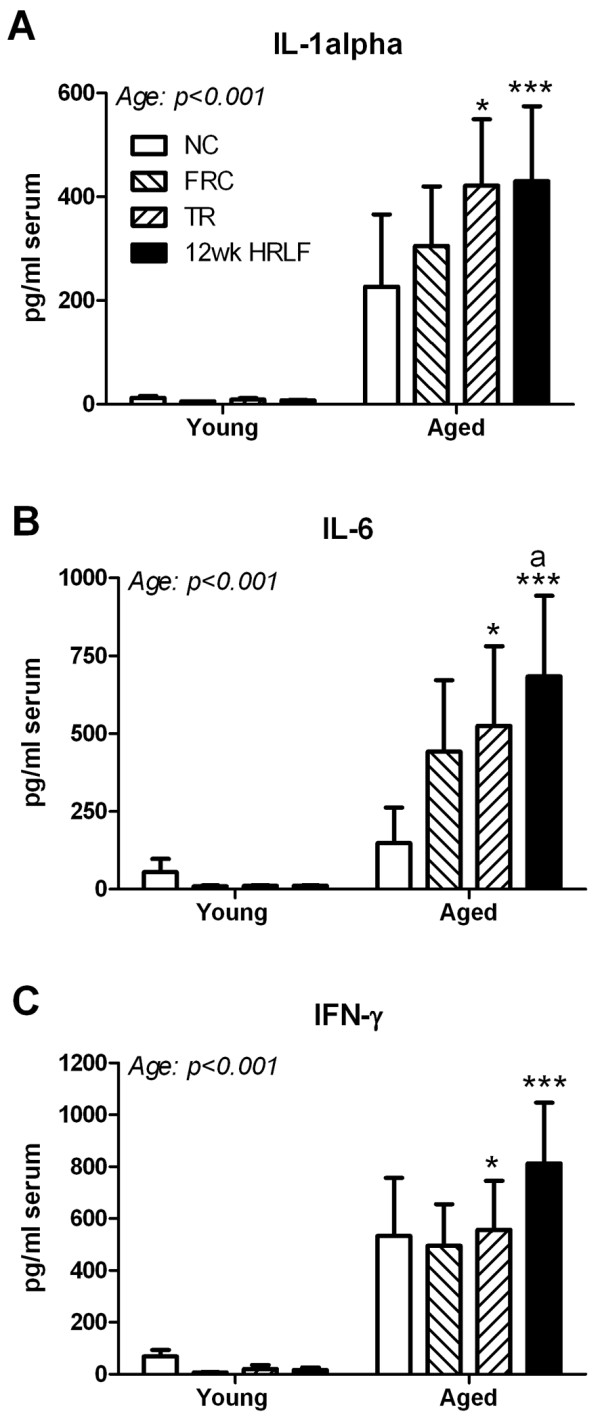
**Three serum cytokines and chemokines, IL-1α (A), IL-6 (B) and IFN-γ (C) increased in trained-only (TR) and high repetition low force task (HRLF) aged rats compared to Trained and HRLF young rats, respectively**. B) IL-6 also increased in aged HRLF rats compared to aged normal controls (NC). Mean + SEM shown. FRC = Food restricted controls that were neither trained nor performed the task. *p < 0.05, and ***p < 0.001 compared to same group of young rats; ^a^p < 0.05 compared to aged NC rats.

We observed significant differences by age, with higher levels of IL-1α, IL-6, and IFN-γ in aged rats than young rats (p < 0.001 each; Figure 2). Significant post hoc results show that IL-1α was increased in aged Trained and aged 12-week HRLF rats, compared to young Trained and young 12-week HRLF rats (p < 0.05 and p < 0.001, respectively; Figure 2A). Similar results were found for IL-6 and IFN-γ (Figure 2B, C). Furthermore, IL-6 was increased in aged 12-week HRLF rats, compared to aged normal controls (p < 0.05, Figure 2B). In contrast, no significant increases in serum cytokines were observed in young 12-week HRLF rats (Figure 2A-C). No significant serum increases were observed as a consequence of food restriction (FRC) compared to same age normal controls of either age group.

No significant serum increases were found for TNFα or MIP2 for either age group (data not shown). IL-10 was below detectable levels with the Assay Gate system used in all but 4 rats (data not shown). Therefore, IL-10 was also tested using the Aushon Searchlight system, which was a more sensitive assay for this analyte. Although detectable using the Aushon Searchlight system, no significant serum increases was observed for IL-10 in any task group compared to same age normal controls or between the two age groups (data not shown).

**Figure 3:**
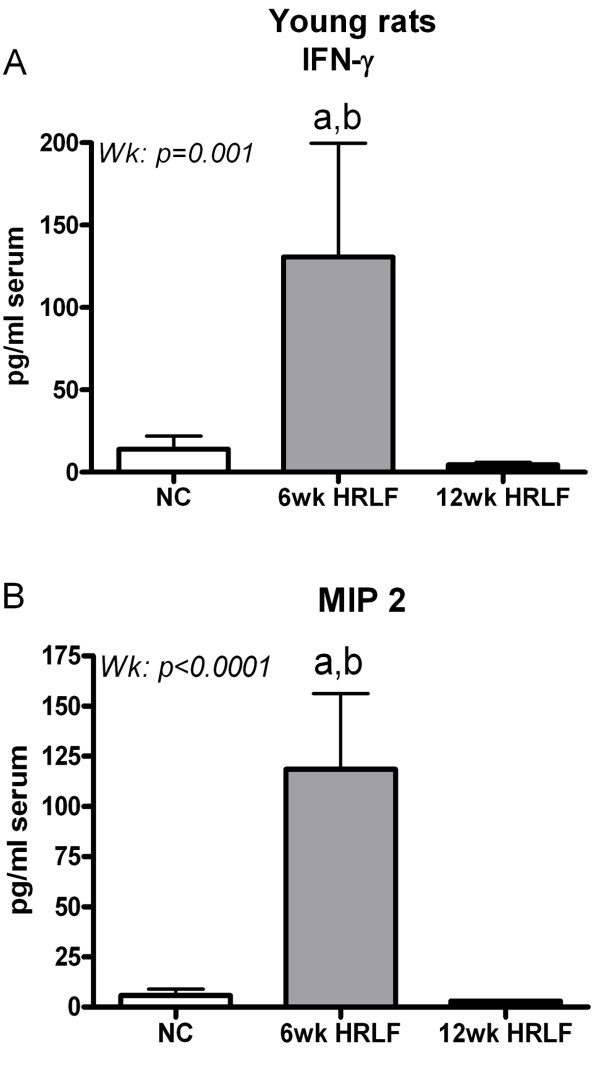
**Two serum cytokines and chemokines, IFN-γ (A) and MIP 2 (B) increased in young 6-week HRLF rats, compared to young normal control rats (NC)**. This increase was absent in young 12-week HRLF rats. Mean + SEM shown. ^a^p < 0.05 compared to young normal control (NC) rats; ^b^p < 0.01 compared to young 12-week HRLF rats.

Since we have previously shown a transient increase of several inflammatory cytokines in serum in a past study in which we were examining the effects of performing a high repetition, negligible force task [13,18], we also examined serum cytokine levels in young rats that had performed the HRLF task for only 6 weeks in order to determine if we had missed their increase by examining only young 12-week HRLF rats. Figure 3 shows a significant increase in IFN-γ (Figure 3A) and MIP 2 (Figure 3B) in young 6-week HRLF rats, compared to young normal controls (p = 0.02 and p = 0.003, respectively). In contrast, there was a significant decline in IFN-γ and MIP 2 in young 12-week HRLF rats, compared to young 6-week HRLF rats (p = 0.001 and p < 0.0001, respectively; Figure 3A, B).

### Grip strength declined with HRLF task performance in both aged and young rats

**Figure 4:**
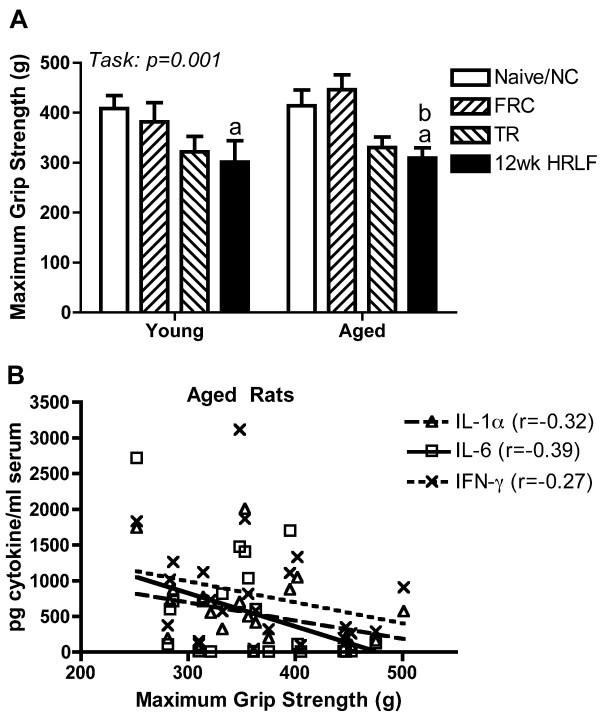
**Grip strength**. (A) Effects of food restriction (FRC; food restricted controls), training (TR) and high repetition low force task (HRLF) performance on maximum grip strength (grams, g) in young and aged rats. (B) Graph showing results of Pearson's correlation tests between grip strength and serum cytokines in aged rats. Mean + SEM shown. ^a^p < 0.05 compared to same age of NC rats; ^b^p < 0.05 compared to same age of FRC rats.

For grip strength, there were significant differences by group (p = 0.001), but not by age (p = 0.36). As shown in Figure 4A, post hoc analysis showed that both young and aged rats that had performed the HRLF task for 12 weeks had significantly decreased grip strength compared to their naïve levels or to normal controls rat levels (p < 0.05 each, thus this data is combined for Figure 4A). Also, aged 12-week HRLF rats had significantly lower grip strength than aged FRC rats (p < 0.05). Pearson's correlation analysis showed a significant negative correlation between serum IL-6 levels and grip strength declines in aged rats (r = -0.39, p = 0.03). The relationship between serum IL-1α and IFNγ levels was also inverse, although there was no significant correlation (r = -0.32, p = 0.06; and r = -0.27, p = 0.10, respectively). Grip strength did not correlate with serum cytokine levels when both aged and young rat data was combined due to the large number of young rats with no increase in serum cytokines (data not shown).

## Discussion

In this study, we found that not all of our initial hypotheses were supported. Aged animals had higher serum levels of two pro-inflammatory cytokines, IL-6 and IFN-γ, as well as increased IL-6, a proteic cytokine with both pro- and anti-inflammatory properties [34], compared to young rats, regardless of group. Also, aging contributed to an increase in IL-6 in aged 12-week HRLF rats compared to aged normal controls, a response missing in young 12-week HRLF rats. These findings are indicative of an enhanced serum cytokine response in aged rats performing a repetitive motion task compared to young rats performing the same task. An increased serum cytokine response (IFN-γ and MIP2) observed in young 6-week HRLF rats was absent in young 12-week HRLF rats, despite the continued performance of the task by, suggestive of a resolution of serum inflammatory response in this age group. We observed that grip strength was equally lower in 12-week HRLF rats of both age groups, compared to normal control and naïve levels. Lastly, grip strength correlated moderately with serum IL-6 levels in the aged rats, but not in the young rats. Thus, aging was a co-factor to a repetitive task-induced serum inflammatory response, but grip strength was apparently affected by factors other than circulating levels of pro-inflammatory cytokines.

### Inflammatory cytokines and aging

Even though the effects of aging on the levels of inflammatory cytokines have some inconsistent results [38], a wealth of data indicates that tissue and serum levels for several inflammatory cytokines, IL-α or β, IL-6, IFN-γ and TNF-α, increase with age [28-33,39-44]. Although these increases may result at least partially from coincident age-associated diseases, increases in circulating inflammatory cytokines have also been demonstrated in older individuals lacking any apparent illness [28,41]. In people, serum IL-6 and TNFα production by peripheral blood mononuclear cells increase exponentially with age until the seventh decade and is higher in women within 10 years of menopause [39,41]. Similar results have also been seen in "normal" (i.e. non-disease-prone) strains of mice and rats as they age [29,33,40]. In aged rodents, serum IL-6 and TNF production increase in response to lipopolysaccharide (LPS) injection, as well as spontaneously, presumably due to altered regulation of their production [33,40]. It is possible that the age-associated rise in inflammatory cytokines is of physiological consequence, rendering an individual susceptible to the myriad of processes induced by these cytokines, including lymphoproliferation, osteoporosis, and a metabolic change towards increased catabolic processes [28,42,45].

Our serum cytokine results in the aged control rats are in agreement with many of the above mentioned studies. In a recent population-based study, Stowe [30] reported that IL-6 and TNF receptor 1 (TNF-r1) significantly increased with age, whereas IL-10 did not change. Although their IL-6 results were confirmed in this study, they used TNF-r1 as a surrogate marker for TNFα due to its better detectability and longer half-life. Therefore, TNF-α finding in this current study may be a false negative result. On the other hand, our unchanged TNF-α level are supported by other publications examining healthy elderly people in which no increases in this cytokine were observed [38,41]. Taken together, our observed significant increases in pro-inflammatory cytokines (IL-1α, IFN-γ) and a proteic cytokine known to lose its regulatory control with age (IL-6) in aged control rats, combined with no significant increase in an anti-inflammatory cytokine (IL-10), may reflect previously reported imbalances in the cytokine network as a result of aging [30]. If so, this may predispose aged subjects to more risk with prolonged repetitive tasks as discussed further below.

### Inflammatory cytokines, repetitive motion tasks and aging

Although we discussed in the previous section that there is a general increase of inflammatory cytokines with aging, in young individuals, increases of serum inflammatory cytokines are typically associated with physiological stress [32]. Their presence in serum has been implicated in the pathogenesis of infection, trauma, muscle catabolism, muscle overuse, cancer, osteoporosis, for example [26,33,42,45]. Cytokines are important molecular messengers in the response of soft tissue to acute injury and wound healing, and are typically tightly regulated since the release of pro-inflammatory cytokines into the extracellular matrix whether during the acute or chronic phases of inflammation can stimulate local and systemic immune reactions. We have observed increased inflammatory cytokines (IL-6, TNF α and IL-1β) in sera of patients with early onset of moderate to severe symptoms of upper limb repetitive strain injury [10], presumably as a result of increased cytokines release in injured or inflamed tissues. These cytokine increases correlated positively with a composite score of increased symptoms of repetitive strain injury derived from an upper body musculoskeletal assessment. This assessment score included declines in grip strength, presence of painful trigger points, and presence and duration, frequency and intensity of pain [10]. In our rat model, we have reported dose-dependent increases of many serum pro-inflammatory cytokines (IL-1α, TNFα, MIP2, MIP3a, RANTES) and an anti-inflammatory cytokines, IL-10 (although only with a high repetition, high force task), in young rats performing a variety of repetitive tasks. Their increases were associated with dose-dependent nerve and musculoskeletal tissue injury, and dose-dependent increases of inflammatory cytokines in nerves, muscles, tendons, bones and even spinal cord [11-13,18,19,22,25]. These injuries and associated increases in tissue cytokines are most likely the source of the serum cytokines in our task rats. However, increased serum cytokines were not found in a primate study by Sommerich and colleagues examining the effects of performing a repetitive pinching task, despite findings of median neuropathy with significant declines in nerve conduction velocity (25-31%) and declines in performance abilities in several animals [46]. Freeland and colleagues also found an absence of inflammatory cytokines (IL-1 and IL-6) in patients with idiopathic carpal tunnel syndrome and abnormal electrophysiological findings at the time of carpal tunnel release surgery, although they found increased IL-6 in biopsied tendosynovial tissues [47].

The results of these last two mentioned studies help explain our young rat results in which we found no increase in serum inflammatory cytokines by week 12 of task performance. In this study, we examined the effects of a moderate demand task at a later time point than we have previously examined in young animals. In prior studies, we have shown that young animals performing a HRNF task (high repetitive, negligible force; 8 reaches/min; < 5% maximum pulling force) had significantly increased serum cytokine levels at 8 weeks [13], but resolution of most tissue macrophage and cytokine responses between 5 and 6 weeks of task performance [11,12,14]. These prior studies indicate that the local musculoskeletal and nerve inflammatory response resolves earlier than the serum cytokine response in young rats performing a moderate demand repetitive task. Here, we observed only a transient increase of two pro-inflammatory cytokines in sera of 6-week HRLF young rats, an increase that was absent by week 12 of task performance. These results combined with those by Sommerich et al [46] and Freeland et al are suggestive [47] of only a transient or even an absent serum cytokine response, dependent on several variables, such as the type of inducing task, the involved tissues, the timing of serum collection, such as before, during or after an acute injury phase or even the age of the individual or animal.

In the aged rats, only IL-6 was increased in the serum of 12-week HRLF task rats compared to age-matched controls. IL-6 is unique as it is a highly pleiotropic cytokine with both pro- and anti-inflammatory properties [34,38]. Its increase in sera of aged HRLF rats compared to age-matched normal controls, and the absence of IL-6 in the serum of young HRLF rats, may indicate that the tissue injury caused by the HRLF task in the aged rats was severe enough to provoke the anti-inflammatory protection mechanism, although the lack of detectable levels of IL-10 limits this hypothesis considerably. While it is impossible to rule out all confounders even in an animal study, we were able to rule out several confounders that can increase serum cytokine levels, such as the loss of regulatory effects of estrogen in post-menopausal mammals or a general increase in sarcopenia due to aging (by inclusion of aged-matched normal controls), existence of detectable cancers or illness (by eliminating rats with detectable presence of these conditions, see methods), the acute effects of exercise (by collecting the serum at 18 hours after the final task period) and any effects induced by the 5% food restriction (by inclusion of age-matched food-restricted controls). We hypothesize that the increased IL-6 in the aged HRLF task rats may be the combined consequence of an aged-related loss of regulation of IL-6, IL-1α and TNFα (indicated by their increased presence in the aged rats) and a decline in tissue healing abilities that prevented tissue adaptation or repair. In our recent study examining the effects of HRLF performance in aged rats [19], we observed median neuropathy that was greater than that seen in our past studies on young rats, even studies examining the consequence of a high force task rather than this moderate demand task [15,16,18,20]. There was even a sustained median neuropathy in the trained-only rats despite a 12 week rest break before euthanasia [19]. This further supports our hypothesis of lowered adaptation or repair of injured tissues in aged rats.

Concerning the serum findings in young versus aged rats, the animals were matched by type of task and its duration. Also, the reach rate exposure was equivalent between the groups and did not contribute to the serum differences detected. These findings partially support those from epidemiological studies demonstrating a relationship between advancing age and increased risk for musculoskeletal disorders [3-6], although more investigation of the tissue changes is needed in these animals for full confirmation of this hypothesis. Furthermore, our findings are limited to an enhancement of the serum cytokine response with age, since grip strength declines were similar after task performance in both ages, as discussed further below.

### Grip strength, repetitive task performance and aging

Our findings of a relationship between grip strength declines and increases in IL-6 in the aged 12-week HRLF rats are consistent with results of several other studies. Serum levels of IL-6 have been suggested to be an independent marker of handgrip and muscle power in aged individuals [48]. The correlation results in the aged rats in this study are similar to prior studies from our own lab in which we have previously found an inverse correlation between increasing serum TNFα, MIP2 and MIP3 levels and grip strength in young rats performing a HRNF task for 8 weeks [13]. Interestingly, a number of studies suggest that high levels of pro-inflammatory cytokines may contribute to an acceleration of sarcopenia (a loss of muscle with age), fragility and other types of tissue catabolism [32,33,42,45]. In one study on mice, lowering serum IL-6 levels experimentally lowered secondary effects in tissues occurring as a consequence of age-associated increases in this catabolic cytokine [33].

However, although grip strength has been used as a reliable biological marker of aging [49,50], our current results show that aging itself did not result in additional motor declines, as indicated by the equivalent grip strength levels in aged versus young rats. Perhaps our aged Sprague Dawley rats were not old enough for additional declines compared to young rats, since they were aged (14-18 mo of age) but not senescent (≥24 mo of age). It may also be that the food restriction, albeit only 5%, slowed tissue catabolism in our aged animals as suggested by several studies [35-37].

The grip strength declines in the young rats combined with the absence of a serum cytokine response indicate that other factors can also affect grip strength. We have observed declines in grip strength in young rats as a result of performance of a repetitive task in other studies from our lab [13,15,19,21,25]. Myofiber fray or degeneration, tendon degenerative changes, and even pathological bone formation at sites of muscle attachment to the bone were observed, each of which might alter the biomechanical properties of these tissues and thus grip strength [12,14,21-24], although several of these changes were induced only with performance of high repetition, high force tasks. We have also observed strong correlations between grip strength declines and muscle (r = -0.72) and tendon (r = -0.85) inflammatory cytokines, and even increased nociceptor-related neurotransmitters in spinal cord dorsal horns (r = -0.70) in young rats performing a high repetition, negligible force task for 8-9 weeks [17,25]. Motor weakness induced as a consequence of nerve dysfunction may also contribute to the observed declines in grip strength. We have observed significant declines in median nerve conduction velocity in young rats performing a HRNF task for 9-10 weeks [16], and in aged rats performing the HRLF task for 12 weeks [19], indicative of nerve dysfunction. The grip strength declines in the latter study correlated strongly with nerve conduction velocity declines (r = -0.70), and moderately with increased nerve and muscle cytokine production (r = -0.38 and r = -0.41, respectively) [19].

## Conclusion

We found that aged rats had higher circulating pro-inflammatory cytokines and chemokines than young rats, supporting our initial hypothesis of an enhancement of the cytokine response with age. Furthermore, performance of repetitive upper extremity task resulted in increased levels of IL-6 in aged HRLF rats compared to aged control rats, further supporting our hypothesis of task-induced increases in serum cytokines. We also observed a transient increase in serum pro-inflammatory cytokines in young HRLF task rats that resolved by week 12, indicative of resolution of the serum cytokine response in the young rats despite continued task performance. Since this resolution was absent in aged HRLF rats, we suggest that there is a loss of tissue adaptation or repair in the aged rats. The increase in serum IL-6 correlated with declines in grip strength in the aged rats. This combined with the enhanced serums response is suggestive of age-related tissue declines that were enhanced by long-term performance of a repetitive task. Surprisingly though, we found that grip strength was also decreased in young HRLF task rats, indicating that grip strength is affected by factors other than systemic inflammation with long-term performance of repetitive tasks.

## List of abbreviations

FRC: food-restricted controls; HRLF: high repetition, low force; IL-1α: interleukin-1alpha; IL-6: Interleukin 6; MIP2: macrophage inflammatory protein 2; MIP3: macrophage inflammatory protein 3; NC: normal controls; TNFα: tumor necrosis factor alpha; TR: trained controls.

## Competing interests

The authors declare that they have no competing interests.

## Authors' contributions

DLX participated in the design of the young rat part of the study, performed the data analyses and drafted the manuscript. MH was responsible for animal care and operant behavioral experiments. CKW performed the grip strength testing. MA participated in the design of the study and in the operant behavioral experiments, and coordinated the serum testing. AEB participated in the design and coordination of the aged rat part of the experiments, and was responsible for the design and construction of the behavioral apparati. MFB conceived, designed and coordinated all aspects of the study, participated in the data analysis, and helped to draft the manuscript. All authors read and approved the final manuscript.

## Pre-publication history

The pre-publication history for this paper can be accessed here:

http://www.biomedcentral.com/1471-2474/12/63/prepub
